# Investigation of Essential Oil from Cumin (*Cuminum cyminum*) Seeds and Selected Terpenes as Repellents Against Adult Female *Phlebotomus papatasi* (Scopoli) (Diptera: Psychodidae) Sand Flies

**DOI:** 10.3390/insects16060599

**Published:** 2025-06-06

**Authors:** Maia Tsikolia, Panagiota Tsafrakidou, Michael Miaoulis, Andrew Y. Li, Dawn Gundersen-Rindal, Alexandra Chaskopoulou

**Affiliations:** 1 European Biological Control Laboratory, USDA-ARS, 54 Marinou Antipa Str., 57001 Thessaloniki, Greece; mtsikolia@ars-ebcl.org (M.T.); panag.tsafrak@gmail.com (P.T.); mmiaoulis@ars-ebcl.org (M.M.); 2 American Farm School, 54 Marinou Antipa Str., 57001 Thessaloniki, Greece; 3 Invasive Insect Biocontrol and Behavior Laboratory, USDA-ARS, 10300 Baltimore Avenue, Beltsville, MD 20705, USA; andrew.li@usda.gov (A.Y.L.); dawn.gundersen-rindal@usda.gov (D.G.-R.)

**Keywords:** GC-MS analysis, cumin aldehyde, spatial repellency, contact repellency, transfluthrin, DEET, octanol, 1-octen-3-ol

## Abstract

Leishmaniasis, a parasitic disease transmitted by sand flies, poses a significant global health threat. Chemical repellents and insecticides are widely used for protection, but prolonged use has led to resistance, reduced efficacy, and environmental concerns, emphasizing the need for new repellent compounds, ideally from sustainable sources. This study investigated the chemical composition and repellent properties of cumin seed essential oil (EO) from Greece against *Phlebotomus papatasi*. Gas chromatography–mass spectrometry (GC-MS) identified five major constituents, including cumin aldehyde (27.0%), *β*-pinene (11.4%), and *γ*-terpinene (10.8%). In addition to cumin seed EO and its major constituents, octanol, and 1-octen-3-ol were tested for comparison, along with transfluthrin and DEET as standard repellents. Using a static air repellency bioassay, cumin seed EO, cumin aldehyde, and octanol exhibited strong spatial repellency (EC_50_ of 0.34, 0.07, and 0.60 μg/cm^2^ respectively) comparable to transfluthrin (EC_50_ of 0.04 μg/cm^2^) at 1 h, and contact repellency, both lasting up to 3 h. This is the first study to evaluate cumin seed EO and cumin aldehyde against sand flies, highlighting their potential as alternatives to conventional repellents. Further research is needed to explore their applicability in vector control strategies.

## Simple Summary

Leishmaniasis, a disease transmitted by sand flies, remains a major global health concern. While chemical repellents and insecticides are commonly used for protection, their prolonged use has led to reduced efficacy, resistance, and environmental concerns. This study analyzed the chemical composition of cumin seed essential oil (EO) from Greece and tested its repellent properties against the sand fly *Phlebotomus papatasi*. Gas chromatography–mass spectrometry (GC-MS) identified five major compounds, including cumin aldehyde, *β*-pinene, and *γ*-terpinene. The repellency of cumin seed EO, its major constituents, octanol, and 1-octen-3-ol was evaluated alongside the established repellents transfluthrin and DEET. Results showed that cumin seed EO, cumin aldehyde, and octanol demonstrated strong repellency, with effects lasting up to 3 h. Cumin aldehyde exhibited repellent activity comparable to transfluthrin, while knockdown effects were observed at higher concentrations. This study is the first to assess cumin seed EO and cumin aldehyde as potential alternatives to conventional repellents for sand flies.

## 1. Introduction

Phlebotomine sand flies (Diptera, Psychodidae) are small insect vectors of several human and animal pathogens, including viruses, bacteria, and most importantly, *Leishmania* species. These protozoan parasites cause leishmaniasis, a neglected tropical disease, which is endemic in more than 90 countries and territories across four continents [1,2,3]. Leishmaniasis control relies heavily on the management of vector populations since human preventive vaccines are not yet available, and existing antimonial treatments have toxic side effects, imposing significant costs for individuals in low-income countries, and are often hindered by poor patient adherence to treatment regimens [4,5]. Sand fly control measures include environmental vector management (EVM) strategies aimed at intervening in their microhabitats to disrupt breeding and resting sites. While these measures can substantially reduce sand fly populations, particularly when integrated with other leishmaniasis elimination methods, they are insufficient as a standalone approach due to the need for strong community awareness and participation. Consequently, chemical interventions (targeted at adult sand flies) are essential as effective complementary strategies to further mitigate the problem [6].

Among chemical interventions aiming to prevent sand fly bites, topical and spatial repellents provide an important layer of personal protection. Spatial repellents offer protection by acting at a distance [7], while topical repellents are applied to the skin or clothing to directly prevent bites from blood-feeding arthropods. Widely used topical repellents such as N,N-diethyl-m-toluamide (DEET), N-(2-methylpiperidin-1-yl)cyclohex-3-ene-1-carboxamide (picaridin), and ethyl butylacetylaminopropionate (IR3535) have demonstrated effectiveness against various insects, including sand flies (e.g., *Phlebotomus* species) [8,9,10,11]. Volatile pyrethroids, such as metofluthrin and transfluthrin, have shown spatial repellent effects against several insect species. For instance, metofluthrin and transfluthrin were proven effective against ticks, including *Dermacentor variabilis*, *Amblyomma americanum*, and *Ixodes scapularis* [12], and transfluthrin has demonstrated efficacy against *Anopheles gambiae* mosquitoes [13].

The efficacy of repellents can differ significantly across the insect species tested. For instance, certain synthetic compounds such as transfluthrin and metofluthrin have shown variable efficacy depending on the environment and the target vector [14,15]. Additionally, growing concerns about the long-term effects of synthetic repellents include potential environmental impacts and the risk of resistance development, particularly in mosquito species like *Aedes aegypti* and *An. gambiae* [16,17,18]. Thus, there is a critical need for research into new repellents that are effective, long-lasting, safe, and capable of targeting a broad range of blood-feeding insects.

Plants produce a range of volatile organic compounds as natural defenses, providing a diverse source of bioactive materials that can serve as direct or structural templates for new control agents [19,20]. Essential oils (EOs) from various plants have demonstrated repellent effects against multiple blood-feeding insects, including sand flies. Specific EOs, such as those from lemon, myrtle, and lemongrass, have shown comparable or superior performance to synthetic repellents like DEET in several sand fly species [21,22,23].

Cumin (*Cuminum cyminum*), a widely used spice in cooking and traditional medicine, is known for its stimulating, tonic, and astringent properties, as well as its antioxidant and antifungal activities [24]. These properties not only enhance the nutritional value of food but also help extend its shelf life. Essential oil from *C. cyminum* seeds has demonstrated pesticidal properties to house flies (*Musca domestica*), green peach aphids (*Myzus persicae*), mosquitoes (*Culex quinquefasciatus*), and moths (*Spodoptera littoralis*), while not affecting non-target invertebrates such as earthworms (*Eisenia fetida*) and aphid predators (*Harmonia axyridis*) [25]. It has exhibited repellent and irritant effects against *An. gambiae* females [26] and larvicidal activity against *Culex pipiens* [27]. Interestingly, an additional bioactivity of cumin seed EO and its main component, cumin aldehyde, is their direct lethal effects against *Leishmania* parasites, indicating their potential as candidates for leishmaniasis treatment [28]. Defined by the FDA as “generally recognized as safe” (GRAS), cumin EO’s insecticidal properties make it a valuable option for organic farming and integrated pest management (IPM) programs [25]. Preliminary data of the cumin seed EO originating in Mexico indicated repellent properties against females of *P. papatasi*, suggesting that this essential oil may be a good candidate for further studies against sand fly vectors.

In this study, we investigated the repellent properties of cumin seed EO and some of its key components against female *P. papatasi*, comparing their efficacy to DEET and transfluthrin (reference standards for contact and spatial repellency, respectively) using a static-air chamber assay. Additionally, we evaluated the impact of two alcohols, 1-octen-3-ol, and octanol, on the behavior of adult female sand flies, both of which are known to elicit species-specific behaviors in insects, e.g., repellency and attractancy [29,30,31,32,33].

## 2. Materials and Methods

### 2.1. Sand Fly Maintenance

The *P. papatasi* colony originated from the Walter Reed Army Institute of Research (WRAIR, Silver Spring, MD, USA) and has been maintained at the European Biological Control Laboratory (EBCL) in Thessaloniki, Greece, since 2020, following the WRAIR protocol from the Biodefense and Emerging Infections Research Resources Repository (BEI) [34]. Adult female *P. papatasi* were provided with 30% sucrose solution daily and maintained in plexiglass cages at an ambient temperature of 26 °C and relative humidity of 80% until used in experiments.

### 2.2. Chemical Sources

Cumin seeds harvested in Greece were sourced from Herbstore.gr (Veria, Greece). The following compounds were obtained from Sigma-Aldrich (St. Louis, MO, USA): *α*-pinene (98% purity), *β*-pinene (99%), *β*-myrcene (88.5%), *p*-cymene (97%), *γ*-terpinene (97%), cumin aldehyde (98%), octanol (97%), 1-octen-3-ol (98%), transfluthrin (>98%), and DEET (97%). Anhydrous Sodium Sulfate (≥99%) was purchased from Honeywell Fluka (Seelze, Germany). Acetone (99.5%) was purchased from Centralchem (Bratislava, Slovakia).

### 2.3. Extraction of Cumin Seed EO (Hydrodistillation)

**Figure 1:**
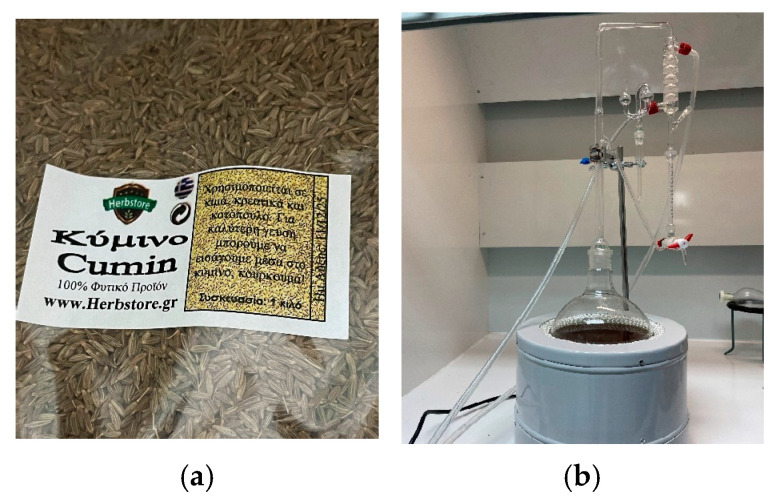
(**a**) Cumin seeds sourced from herbstor.gr; (**b**) hydrodistillation of cumin seeds conducted with a Clevenger-type apparatus.

Cumin seeds (300 g) were placed in a round-bottom flask (2000 mL) and covered with deionized (DI) water (~1000 mL) (Figure 1). The temperature was set to ~100 °C, while the water-cooling system was set on a high stream. After 45 min of heating, the mixture began to boil, initiating the distillation process. The distillation continued for 3 h, after which time the heating was stopped, and the system was allowed to cool down [35]. The obtained oil was dehydrated over anhydrous sodium sulfate and stored in a sealed amber glass vial at 4 °C until further use.

### 2.4. Gas Chromatography–Mass Spectrometry (GC–MS) Analysis with Electron Ionization (EI)

A GCMS-QP2020 (Shimadzu, Columbia, MD, USA) system with an EI detector, equipped with an SH-Rxi-5ms capillary column (30 m length, 0.25 mm i.d., and 0.25 μm film thickness; Shimadzu, Columbia, MD, USA), an AOC-6000 Plus autosampler and autoinjector (Shimadzu, Columbia, MD, USA) was utilized to analyze the cumin seed EO. The injector, ion source, and interface temperatures were maintained at 250 °C, 260 °C, and 240 °C, respectively. The GC oven temperature program was set at an initial temperature of 35 °C, held for 10 min post-injection, then ramped at 3 °C/min to 100 °C and held for 10 min. It was further ramped at 4 °C/min to 200 °C, held for 5 min at the same temperature, and finally reached 240 °C (held for 30 min) at a 5 °C/min pace. Helium was used as the carrier gas at a constant flow of 1.17 mL/min, with a split ratio of 1:30, pressure at 62 kPa, and a linear velocity of 39.0 cm/s at 35 °C. Mass spectra were recorded at 70 eV. Data acquisition was conducted using GCMS solution software v. 4.52 (Shimadzu, Columbia, MD, USA). The EO solution in acetone was injected into the column (liquid injection). The injection volume was 1 µL.

Compound identification: The components were identified by comparing their relative retention times to those of authentic standard compounds or by comparing their relative retention indices (RRI, Kovats retention indices) to a series of C_6_-C_30_ n-alkanes [36]. Compounds were analyzed under the same conditions and compared with literature data. Non-isothermal Kovats retention indices were calculated using the equation:

RRIx = 100 n + 100 (tx − tn)/(tn + 1 − tn)(1)

where tn and tn + 1 are the retention times of the reference n-alkanes eluting before and after compound “X”, and tx is the retention time of compound “X”. Mass spectra were compared to those of standard compounds and searched against the NIST (US National Institute of Standards and Technology, Gaithersburg, MD, USA) and FFNSC 2 (Flavour & Fragrance Natural & Synthetic Compounds, Shimadzu Corp., Kyoto, Japan) GC-MS libraries for identification.

### 2.5. Static-Air Repellency Bioassay

**Figure 2:**
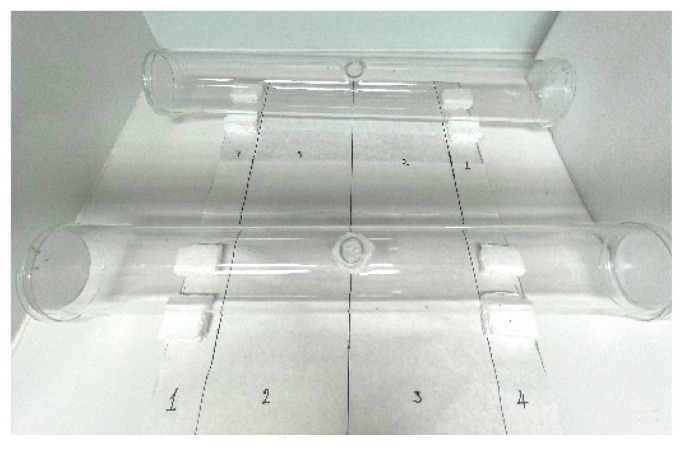
Static-air chamber used in repellency bioassays.

The bioassay was adapted from Paluch et al. [37] and Temeyer et al. [11]. All experiments were conducted under controlled ambient conditions of 26 ± 1 °C in static air chambers (60 cm in length and 9 cm outer diameter glass tubes) placed inside a fume hood. Illumination was provided by overhead room lighting from white LED bulbs, while the fume hood’s internal light was kept off to ensure consistent conditions. One ml of test solution (acetone + active ingredient) or solvent alone (acetone) was applied on 9 cm (63.6 cm^2^) Whatman No. 1 filter paper disks (Sigma-Aldrich, St. Louis, MO, USA) and left to air dry until the acetone had completely evaporated (~10 min). Filter papers were then placed in glass Petri dish lids that were used to seal the opposite ends of the tubes. Each treatment tube contained one untreated (solvent alone) and one treated (test solution) filter paper, while for the control tubes, two untreated filter papers were used. Adult female (groups of 20 ± 3) *P. papatasi* sand flies (3–7 days post-emergence, not blood-fed) were released in the middle of the glass tube (via a circular opening) using a mouth aspirator. Prior to insect release, the tubes were divided into 4 equal sections to allow for accurate recording of insect location across the tube gradient (Figure 2). The distribution of sand flies across the tubes and on the filter papers was recorded at 15, 30, 60, 90, 120, and 180 min post-release.

Repellent activity (spatial and contact) was assessed using concentrations that did not cause an insect knockdown effect within 2 h of exposure. Knockdown was defined as the insects’ partial paralysis and inability to (a) maintain an upright posture and (b) fly along the static-air chamber [38]. After completing the experiments, the glass tubes and Petri dishes were washed with soap and rinsed with acetone and DI water. To ensure complete removal of highly active compounds such as transfluthrin, an additional step of overnight baking at 180 °C was performed. Each treatment assay was performed in four to seven replicates, while the control assays were conducted at least seven times.

Test solutions were prepared in acetone [weight/volume (*w*/*v*) %] and stored in 15 mL amber vials. The initial concentrations tested were 157.2 μg/cm^2^ (prepared from a 1% solution) for cumin seed EO, and 78.6 μg/cm^2^ (from a 0.5% solution) for all substances except transfluthrin. If knockdown occurred during the bioassay, concentrations were lowered until no toxic effects were observed within the first 2 h. Cumin seed EO and cumin aldehyde were also tested at lower concentrations of 39.2 and 19.6 μg/cm^2^ (from 0.25% and 0.125% solutions, respectively). Transfluthrin was tested at 7.86, 0.786, 0.1572, and 0.0786 μg/cm^2^ (from 0.05%, 0.005%, 0.001%, and 0.0005% solutions, correspondingly). Additionally, active substances and standard repellents were further tested at lower concentrations to estimate the half-maximal effective spatial repellency concentration (EC_50_). Half-maximal effective concentration is defined as the concentration of a compound which induces a repellency effect in 50% of the test insects after a specified exposure time. The concentrations tested for each compound, along with the corresponding formulations, are provided in Supplementary Table S1.

### 2.6. Data Analysis

All statistical analyses were conducted using R Statistical Software (version 4.3.0; R Core Team, 2023) [39] within RStudio [40]. Additionally, EC_50_ values were cross-referenced using AAT Bioquest’s EC_50_ calculator [41]. A significance threshold of *p* < 0.05 was applied throughout the analysis. Data were analyzed using both conventional parametric methods and mixed-effects statistical approaches, as appropriate. Prior to analysis, outliers were removed. For parametric analyses, assumptions of normality and homogeneity of variance were tested using the Shapiro–Wilk and Levene’s tests, respectively.

Following the methods described by Paluch et al. [37] and Temeyer et al. [11], spatial repellency was calculated at each of the six time points during the bioassay using the formula:

Repellency (%) = [(Nu − Nt)/N] × 100(2)

where Nu represents the number of insects located on the untreated half portion of the tube (with the untreated filter paper), Nt represents the number of insects located on the treated half of the tube (with the treated filter paper), and N represents the total number of insects released in the tube. The same approach was used for the control tubes, only in this case, one side was randomly assigned as “treated” and was constantly rotated across the experiments. A one-way Analysis of Variance (ANOVA) was performed, followed by post hoc Dunnett’s test for multiple comparisons to compare the spatial repellency (%) between each treatment and the control at each time point (15, 30, 60, 90, 120, and 180 min). To evaluate temporal changes within each treatment, linear mixed-effects models (LMMs) were applied using the Kenward–Roger method, with time as a fixed effect and replicate as a random effect. Sidak-adjusted post hoc tests were used for pairwise comparisons across time points. Spatial repellency concentration–response curves and EC_50_ values, along with the coefficient of determination (R^2^) and slope, were determined using nonlinear regression with a four-parameter logistic model.

For contact repellency, we calculated the avoidance frequency of the treatment by measuring how frequently at least one out of twenty (±3) sand flies touched or rested on the treated filter papers at each of six time points during the bioassay. The same approach was used for the control tubes, only in this case, one side was randomly assigned as “treated” and was constantly rotated across the experiments. A score of ‘1’ denoted complete avoidance of the filter papers by all flies (i.e., no flies landing on the surface), while a score of ‘0’ indicated that at least one fly made contact [37,42]. Mean avoidance frequency was determined by averaging the scores across the six time points (15, 30, 60, 90, 120, and 180 min), reflecting the overall avoidance behavior throughout the 3 h experiment. Additionally, following the approach described by Paluch et al. [37], statistical comparisons were made by computing the mean avoidance ratios (%) of sand flies in the treated versus the control tubes across the six-time observation points; *p*-values were calculated using a two-tailed Fisher’s Exact test. The mean avoidance ratio (%) was computed for all treatments and blank controls using the formula:

Mean avoidance ratio (%) = Nt × 100/(Nt + Nu)(3)

where Nt and Nu represent the number of insects on treated and untreated filter papers, respectively (for blank controls, a side was randomly assigned as “treated” and was constantly rotated across experiments).

Knockdown percentage (KND (%)) was calculated using the formula:

KND (%) = (N_knd_/N_total_) × 100 (4)

where N_total_ is the total number of insects released in the tube, and N_knd_ represents the number of insects exhibiting knockdown effects at observation intervals of 15, 30, 60, 90, 120, and 180 min. Treatment differences in KND (%) were assessed using one-way ANOVA, followed by Tukey’s honestly significant difference (HSD) post hoc test. ANOVA was applied uniformly, including in cases with only two groups, to ensure methodological consistency across all comparisons. When ANOVA assumptions were violated, the Kruskal–Wallis test was used, followed by Dunn’s post hoc test with Bonferroni correction. Temporal differences within each treatment were analyzed using LMMs, using the Kenward–Roger method, with time as a fixed effect and replicate as a random effect, followed by Sidak-adjusted post hoc tests.

## 3. Results

### 3.1. Chemical Constituents of Cumin Seed EO Revealed by GC-MS

Cumin seed EO was obtained as colorless material in ~2.3% (*w*/*w*, ~6.8 g) yield using the standard hydrodistillation procedure with the Clevenger apparatus. A total of 24 compounds were identified out of 26 peaks in EO, using GC-MS, representing more than ~99% of the total oil composition. The main components (above 4% by relative peak area, ~94% of the total oil composition) were: cumin aldehyde (27.0%), *p*-mentha-1,4-dien-7-al (20.3%), *p*-mentha-1,3-dien-7-al (15.8%), *β*-pinene (11.4%), *γ*-terpinene (10.8%), *p*-cymene (5.1%), and *p*-menth-3-en-7-al (4.1%) (Table 1).

Monoterpenoids accounted for nearly all (~99%) of the identified compounds, with oxygenated monoterpenes representing ~69% and monoterpene hydrocarbons ~30%. Sesquiterpenoids were present in trace amounts, comprising about 0.29% of the EO, with sesquiterpene hydrocarbons (~0.23%) being more abundant than oxygenated sesquiterpenes (~0.06%).

### 3.2. Bioassay Results

#### 3.2.1. Spatial and Topical Repellency Screening for Cumin Seed EO and Other Test Materials Against Adult Female *P. papatasi*

**Figure 3:**
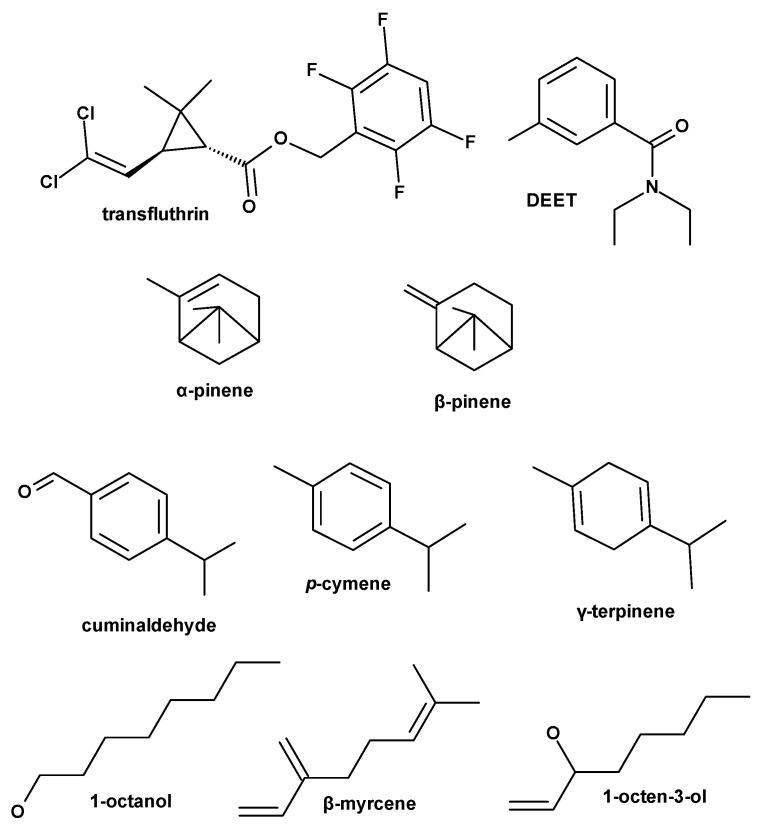
Structures of compounds tested: Tranfluthrin, DEET, *α*-pinene, *β*-pinene, *γ*-terpinene, *p*-cymene, octanol, *β*-myrcene, and 1-octen-3-ol.

Mean spatial repellency (%) of treatments at different time points (15, 30, 60, 90, 120, and 180 min), mean avoidance frequency, and *p*-values for mean avoidance ratios over 3 h vs. controls are summarized in Table 2, while the structures of all compounds tested are shown in Figure 3. As mentioned already, repellency screening test results are provided at concentrations when no knockdown occurred at least within the first 2 h post-treatment exposure. This concentration for most treatments was at 78.6 μg/cm^2^, for cumin seed EO and cumin aldehyde at 19.6 μg/cm^2^, and for transfluthrin at 0.1572 μg/cm^2^ (the latter resulted in high spatial toxicity even at low doses not allowing us to test its repellency effects at concentrations comparable to the rest of the test material in this specific bioassay set-up) (Table 2).

##### Spatial Repellency

Compared to controls, *p*-cymene, octanol, 1-octen-3-ol, cumin seed EO, and cumin aldehyde (except at 180 min) demonstrated significant spatial repellency at all time points (ANOVA: 15 min, F_11,34_ = 18.5; 30 min, F_11,34_ = 18.3; 60 min F_11,34_ = 21.1; 90 min F_10,29_ = 15.0; 2 h, F_10,29_ = 18.4; 3 h, F_10,29_ = 11.9; *p* < 0.001; post hoc Dunnett, *p* < 0.05) (Table 2). Cumin seed EO (at 19.6 μg/cm^2^) and octanol (at 78.6 μg/cm^2^) maintained nearly 100% spatial repellency at all observation time points, while cumin aldehyde (19.6 μg/cm^2^) showed a significant decrease in effectiveness at 3 h (LMM: F_5,15_ = 9.8, *p* < 0.001; post hoc Sidak-adjusted, *p* < 0.05) (Table 2). *β*-Myrcene, *p*-cymene, 1-octen-3-ol, and DEET (at 78.6 μg/cm^2^ each) significantly increased in activity from ~14–55% to 75–97% over 2 h (LMM: F_5,10_ = 4.8, F_5,10_ = 4.7, F_5,10_ = 28.5; and F_5,15_ = 9.3; *p* < 0.05; post hoc Sidak-adjusted, *p* < 0.05). For transfluthrin (0.1572 μg/cm^2^), *α*-pinene, and *β*-pinene (78.6 μg/cm^2^), changes in spatial repellency over this period were not significant (LMM: F_5,12_ = 1.4, F_5,15_ = 2.0, and F_5,10_ = 2.3; *p* > 0.05).

##### Contact Repellency

All treatments exhibited significantly different mean avoidance ratios compared to control (Fisher’s Exact 2-tailed test, *p* < 0.05) (Table 2). Regarding avoidance frequencies, *γ*-terpinene, *α*-pinene, *β*-pinene, and *β*-myrcene exhibited the lowest values, ranging from 0 to 0.3, indicating weak contact repellent activity. In contrast, *p*-cymene, octanol, 1-octen-3-ol, cumin seed EO, and cumin aldehyde demonstrated strong contact repellency, comparable to DEET, with avoidance frequencies of ~1.0 over the 3 h period, based on observations at six time points. Transfluthrin also exhibited a high avoidance frequency (0.7) even at the extremely low range of concentrations tested in this bioassay (Table 2).

#### 3.2.2. Determination of EC_50_ Values for Spatial Repellency of Selected Substances Compared to DEET and Transfluthrin

**Figure 4:**
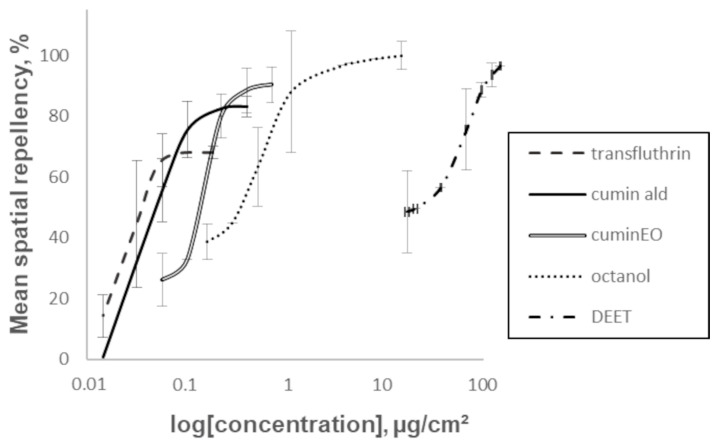
Concentration–response curve for 1 h spatial repellency of cumin seed EO, cumin aldehyde, and octanol against adult female P. papatasi sand flies, compared to transfluthrin and DEET, as measured in a static-air chamber assay. Error bars represent the ±SEM.

Substances that demonstrated significant spatial and contact repellency, namely cumin seed EO, cumin aldehyde, and octanol, were further evaluated to determine their EC_50_ values (Table 3; values are displayed along with 95% confidence intervals (CI), slopes, and R^2^ for each treatment at 15, 30, and 60 min, in comparison to DEET and transfluthrin). Transfluthrin exhibited the lowest EC_50_ values (0.06, 0.03, and 0.04 μg/cm^2^ at 15, 30, and 60 min, respectively) of spatial repellency. In comparison, cumin seed EO, cumin aldehyde, and octanol EC_50_ values were approximately 0.97–0.34, 0.15–0.07, and 1.40–0.60 μg/cm^2^, respectively. DEET exhibited the highest EC_50_ values (~91–79 μg/cm^2^), making it significantly less effective as a spatial repellent compared to the other treatments at all three time points. Most treatments showed stable EC_50_ values over time, except cumin seed EO, which decreased from 0.97 μg/cm^2^ at 15 min to 0.34 μg/cm^2^ at 60 min. Slopes of the concentration–response curves ranged from ~1 to ~26 across treatments and time points, with R^2^ values generally above 0.90 for cumin seed EO, cumin aldehyde, and octanol (Table 3). DEET and transfluthrin had R^2^ values around 0.80, except at 60 min, where they dropped to ~0.71. The predicted sigmoidal concentration–response curves for these repellents are illustrated in Figure 4.

#### 3.2.3. Knockdown Effects of Cumin Seed EO and Cumin Aldehyde Compared to Transfluthrin

During the 3 h static-air chamber assays, cumin seed EO, cumin aldehyde, and transfluthrin at concentrations as low as 78.6, 19.6, and 0.786 μg/cm^2^, respectively, induced knockdown in insects. Other treatments, including DEET, did not induce knockdown at concentrations as low as 78.6 μg/cm^2^.

Knockdown levels (KND ± SEM, %) for cumin seed EO, cumin aldehyde, and transfluthrin are shown in Table 4. At a concentration of 157.2 μg/cm^2^, the knockdown effect of cumin seed EO increased significantly over time, from ~0–8% (at 15 min to 1 h) to ~70% (at 1.5 h), reaching 100% at 2 h; at a lower concentration of 78.6 μg/cm^2^, KND ranged from 0 to 25% (at 15 min to 1 h) to about 96% (at 3 h) (LMM: 157.2 μg/cm^2^, F_5,15_ = 81.4; 78.6 μg/cm^2^, F_5,15_ = 9.1; *p* < 0.001; post hoc Sidak-adjusted, *p* < 0.05). No knockdown effect was observed for cumin seed EO at the lower concentration of 39.2 μg/cm^2^.

For cumin aldehyde, the mean knockdown levels at 78.6 μg/cm^2^ increased significantly from 0% (at 15–30 min) to ~97% (at 3 h), and at 39.2 μg/cm^2^, from 0 to 17% (at 15–90 min) to ~63–99% (at 2–3 h) (LMM: 78.6 μg/cm^2^, F_5,15_ = 6.7; 39.2 μg/cm^2^, F_5,15_ = 24.6, *p* < 0.001; post hoc Sidak-adjusted, *p* < 0.05). Knockdown at the lower concentration of 19.6 μg/cm^2^ was observed only at 3 h (~57%).

Transfluthrin exhibited the greatest toxic effect, with a significant increase in a knockdown from approximately 43% (at 15 min) to 74% (at 3 h) at a concentration of 7.86 μg/cm^2^ (LMM: F_5,15_ = 81.4, *p* < 0.05; post hoc Sidak-adjusted, *p* < 0.05). In contrast, at the lower concentration of 0.786 μg/cm^2^, knockdown levels remained between ~9% and 18%, showing no significant changes.

Significant differences in knockdown levels were observed between different concentrations of treatments at specific time points (Table 4). For instance, cumin seed EO showed a significant reduction in knockdown from 100% to ~31% at 2 h when its concentration was reduced by 50% (from 157.2 μg/cm^2^ to 78.6 μg/cm^2^) (Kruskal–Wallis: χ^2^(1) = 3.9, *p* < 0.05; post hoc Dunn’s test, *p* < 0.05). Similarly, reducing the concentration of cumin aldehyde from 78.6 or 39.2 μg/cm^2^ to 19.6 μg/cm^2^ led to a significant decrease in knockdown from ~64% to 0% at 2 h (Kruskal–Wallis: χ^2^(2) = 7.8, *p* < 0.05; post hoc Dunn’s test, *p* < 0.05). Transfluthrin exhibited a significant reduction in knockdown at all time points starting from 30 min when its concentration was reduced tenfold (from 7.86 μg/cm^2^ to 0.786 μg/cm^2^) (ANOVA: 30 min, F_1,4_ = 41.8, *p* < 0.01; 60 min, F_1,4_ = 26.7, *p* < 0.01; 90 min, F_1,4_ = 48.0, *p* < 0.01; 120 min, F_1,4_ = 14.8, *p* < 0.05; 180 min, F_1,4_ = 125.1; *p* < 0.001; post hoc Tukey, *p* < 0.05).

## 4. Discussion

To explore new compounds for the management of sand flies, we evaluated the repellent properties of cumin (*C. cyminum*) seed EO and its key components, along with 1-octen-3-ol, octanol, DEET, and transfluthrin against adult female *P. papatasi*. Cumin seeds harvested in Greece yielded approximately 2.3% (*w*/*w*) EO through hydrodistillation, consistent with results from previous studies [43,44]. The chemical composition of the EO was dominated by monoterpenoids (~99%), a profile in line with cumin EOs from various geographic origins [24,25,45]. Cumin aldehyde was among the major constituents, as commonly observed in samples from Egypt, Mexico, Turkey, and India [44,46,47,48]. Other prominent compounds included *p*-mentha-1,4-dien-7-al, *p*-mentha-1,3-dien-7-al, *β*-pinene, and *γ*-terpinene, which are also frequently reported in cumin oils [25,48]. An exception in the present sample was phytone, a compound not typically found in cumin EO but known to occur in other plant oils such as those from *Chrysanthemum balsamita* and *Origanum vulgare* [48,49].

In the present study, cumin seed EO demonstrated 100% spatial repellency against adult female *P. papatasi* sand flies at a concentration of 19.6 µg/cm^2^ within 15 min. The estimated EC_50_ was 0.97 µg/cm^2^ at this interval, decreasing to 0.34 µg/cm^2^ after 1 h. Similarly, cumin aldehyde achieved ~97% spatial repellency at the same concentration and time interval. However, its estimated EC_50_ for spatial repellency was considerably lower, reaching 0.15 µg/cm^2^ at 15 min. The versatile effectiveness of cumin seed EO and cumin aldehyde has been documented against various other insect species [24,25,26,27,50]. Deletre et al. [26,50] tested cumin seed EO at a concentration that was approximately 5 times higher than that used in our study (92 µg/cm^2^ vs. 19.6 µg/cm^2^), yet observed lower spatial repellency (~ 37–42%) against adult female *An. gambiae* mosquitoes after 10 min of exposure using a high-throughput screening system (HITSS) set-up. In the same bioassay, cumin aldehyde was more effective against pyrethroid- and organophosphate-resistant *An. gambiae* strains, showing repellency rates of 47–53% at ~29 µg/cm^2^ [18]. The configuration of their system restricted the dispersion of the treatment along the length of the tube. Therefore, besides testing different insect species, the differences observed in our results may be attributed to differences in methodology and experimental design.

It should be noted that, although cumin aldehyde was a more effective spatial repellent than cumin seed EO, based on concentration–response data, its repellency at 19.6 μg/cm^2^ declined markedly over time. Specifically, spatial repellency dropped to ~75% after 1.5 h and further decreased to ~9% after 3 h, when significant knockdown effects were observed. In contrast, the repellent activity of cumin seed EO remained stable at the same concentration. As a complex mixture, the EO contains only 27% cumin aldehyde, along with various other constituents previously discussed. The observed differences may be due to synergistic interactions among the EO’s multiple bioactive components, which could contribute to its sustained repellency. According to Mota et al. [51], compounds with aromatic and polar chemical groups, enabling hydrogen bonding, are crucial for repellent activity. Benelli et al. [25] similarly emphasized the role of aldehyde groups, such as those present in cumin seed EO components like γ-terpinen-7-al and α-terpinen-7-al, in insecticidal properties. Cumin aldehyde, the primary constituent of cumin seed EO, possesses both an aromatic ring and a polar aldehyde group (CH=O), supporting its role in repellency and toxicity. Our findings suggest that cumin aldehyde plays an important role in both repellency and toxicity (knockdown effect) of cumin seed EO.

Given these properties, we further evaluated the knockdown activity of cumin seed EO and cumin aldehyde in comparison with transfluthrin as a synthetic standard. At 78.6 μg/cm^2^, both cumin seed EO and cumin aldehyde induced ~25% knockdown after 1 h, rising significantly to 96% at 3 h. For cumin seed EO, these results are in line with Deletre et al. [26], who reported a 19% knockdown in *An. gambiae* after 1 h of exposure to 92.5 μg/cm^2^ using a WHO test kit. For cumin aldehyde, Deletre et al. [50] reported ~22% knockdown and ~39% mortality at 29 μg/cm^2^ immediately after exposure against *An. gambiae* with the same bioassay. This differs from our findings for *P. papatasi* sand flies at a comparable concentration (39.2 μg/cm^2^), where there was no significant effect until 2 h, but reached 99% knockdown after 3 h. The delayed but strong activity in our case indicates again that bioassay design and insect species significantly influence the observed toxicity. Additionally, transfluthrin, tested at a much lower concentration (7.86 μg/cm^2^), resulted in 60% knockdown at 1 h and 74% at 3 h. These effects are consistent with literature reports of transfluthrin’s high potency for other insect species. Half-maximal knockdown (KC_50_) values of transfluthrin have been shown to range from 0.13 to 5 μg/cm^2^ across different mosquito species and assay formats [38,52,53].

Regarding transfluthrin’s repellency effects, although it has been studied and tested against mosquitoes and other pest insects, up until recently, there were no published data considering its activity as a chemical control method for managing sand flies. Powell et al. [14] evaluated the spatial repellent efficacy of transfluthrin against both mosquitoes and sand flies in open-air and confined space scenarios. Their results suggest that transfluthrin presents species and placement-dependent variability in effectiveness. When used outdoors, it effectively reduced vector populations (although not statistically significantly in the case of mosquitoes), but notably in the enclosed setting, mosquito populations were increased, and its efficacy against sand flies was reduced, reaching a mean repellency of 33.3%. Verhulst et al. [52] also highlighted species-specific sensitivity, showing that transfluthrin at 0.1 µg/cm^2^ was more effective against *Culicoides* midges (50–75% repellency over 15–60 min) than against *Ae. aegypti* (22–50% in the same period). Several other studies have reported EC_50_ values for the spatial repellency of transfluthrin against mosquitoes ranging from 0.06 to 0.8 µg/cm^2^ against various species and exposure times [38,53]. In our results, transfluthrin exhibited strong spatial repellency with EC_50_ values of 0.06, 0.03, and 0.04 μg/cm^2^ at 15, 30, and 60 min. Our initial screenings showed that transfluthrin at 0.1572 µg/cm^2^ displayed spatial repellency rates of 32–23% over 15 to 60 min, averaging 28% over the 3 h period, with higher concentrations resulting in knockdown rather than repellency. This reduced spatial repellency and avoidance frequency observed in higher doses of transfluthrin may be attributed to disorientation effects, similar to those observed in mosquitoes exposed to pyrethroid vapors [14,54]. Such disorientation can impair insects’ ability to correctly recognize or respond to repellents, complicating the interpretation of repellency data [55,56]. The mode of action underlying this influence on behavior by pyrethroids is yet to be unraveled [52].

Octanol was another compound with a high spatial and contact repellent effect against sand flies in our bioassay, albeit in higher doses compared to cumin seed EO and cumin aldehyde. While previously reported as an attractant for New World sand flies, with *Nyssomyia neivai* and *Lutzomyia longipalpis* showing ~70% and ~39% attractancy at 6.25 mg/cm^2^ [31,33], our results indicate that it functions as a repellent for *P. papatasi*. Additionally, Yousefi et al. (2020) [56] observed no attractancy effects of octanol, when used as trap bait, for *Phlebotomus* and *Sergentomyia* sand flies in field experiments conducted in Iran. Octanol has also exhibited repellent effects against different dipteran insect species, such as *Ae. aegypti* and Queensland fruit flies (*Bactrocera tryoni*) [32,57]. These variations highlight that the insects’ physiological state, as well as genetic and behavioral differences among vector populations, affect their responses to chemical stimuli. Experimental factors such as assay design, environmental conditions, and compound formulation or concentration can also affect results. Therefore, in addition to species-specific testing, broad-spectrum and methodologically standardized evaluations are essential to accurately assess the general applicability of repellent compounds in vector control strategies.

Of particular interest are the findings regarding *p*-cymene and 1-octen-3-ol. Although they both showed relatively low (~40–55%) initial spatial repellency, they exhibited strong contact repellency, with avoidance frequencies approaching ‘1’, throughout the 3 h observation period. This is particularly notable for 1-octen-3-ol, which has not previously been described as a repellent for sand flies. Depending on species and experimental context, it has shown attractant, repellent, or neutral effects. The responses of sand flies range from no attractancy in the absence of CO_2_ [58] to weak attraction for *L. longipalpis* [59], and clear attractancy for specific species [33,60,61]. In mosquitoes, its effects are similarly variable, with studies reporting both attractant and repellent properties depending on species and conditions [29,30]. On the other hand, *p*-cymene has documented repellent activity against adult female *C. pipiens* [62] and *M. domestica* [63]. Given the strong contact repellency observed in our assays and their sustained spatial effects over time, both compounds warrant further investigation as potential candidates for long-lasting repellent formulations.

Finally, DEET, tested at 78.6 µg/cm^2^, achieved a mean spatial repellency of ~53% over 3 h of exposure, which falls in the range of previously reported repellency against *P. papatasi* in an identical bioassay set-up [11]. At shorter exposure times (15 min), DEET’s repellency was approximately 25%, notably weaker than transfluthrin and cumin aldehyde. In addition, DEET showed no knockdown effect at 78.6 µg/cm^2^, which is consistent with findings by Verhulst et al. [52] and Deletre et al. [26], who reported little or no knockdown from DEET at concentrations below 100 µg/cm^2^ against *Ae. aegypti* and *An. gambiae*, respectively. These findings, alongside earlier studies on DEET’s varying effectiveness across mosquito species and insecticide-resistant strains [18,26,50,53], underscore the necessity for species-specific testing in repellent development.

In conclusion, this study demonstrates that cumin (*C. cyminum*) seed EO, its main component cumin aldehyde, and octanol exhibit strong spatial and contact repellency against adult female *P. papatasi*, with efficacy comparable to DEET and transfluthrin. Notably, this work is the first to determine EC_50_ values for the spatial repellency of transfluthrin against adult sand flies. It is also the first to evaluate the repellent properties of cumin seed EO and cumin aldehyde against *P. papatasi*, suggesting their potential as natural alternatives to synthetic repellents. Additionally, the effectiveness of cumin aldehyde against pyrethroid- and organophosphate-resistant *An. gambiae* strains [18] highlights its potential to be developed as new repellents against insecticide-resistant sand flies and other disease vectors.

## Figures and Tables

**Table 1:** Chemical composition (by relative peak area, %) of the essential oil (EO) from cumin (*C. cyminum*) seeds sourced from Greece, obtained through Clevenger hydrodistillation and analyzed by GC-MS.

#	Compound	RT	RI	Peak Relative Area, %	Identification
1	*α*-pinene	17.17	929	0.46	RRI, MS, S
2	*β*-pinene	20.041	972	11.45	RRI, MS, S
3	*β*-myrcene	21.44	991	0.93	RRI, MS, S
4	*p*-cymene	23.37	1022	5.14	RRI, MS, S
5	D-limonene	23.55	1025	0.82	RRI, MS
6	1,8-cineol	23.68	1027	0.38	RRI, MS
7	*γ*-terpinene	25.46	1057	10.79	RRI, MS, S
8	trans-sabinene hydrate	27.76	1095	0.15	RRI, MS
9	linalool	28.02	1099	0.14	RRI, MS
10	cis-*p*-menth-2-en-1-ol	29.04	1118	0.06	RRI, MS
11	terpinen-4-ol	32.09	1172	0.38	RRI, MS
12	*α*-terpineol	32.89	1187	0.10	RRI, MS
13	*p*-menth-3-en-7-al	33.08	1190	4.08	RRI, MS
14	cumin aldehyde	36.12	1230	26.98	RRI, MS, S
15	phellandral	38.85	1264	0.44	RRI, MS
16	*p*-mentha-1,3-dien-7-al	39.77	1275	15.83	RRI, MS
17	*p*-mentha-1,4-dien-7-al	40.52	1284	20.27	RRI, MS
18	*p*-mentha-1,4-dien-7-ol	44.16	1333	0.63	RRI, MS
19	RT:44.734	44.71	1340	0.15	RRI, MS
20	RT:46.185	46.19	1361	0.07	RRI, MS
21	1-tetradecene	48.579	1394	0.45	RRI, MS, S
22	trans-*β*-farnesene	51.93	1460	0.04	RRI, MS
23	*β*-acoradiene	52.59	1474	0.19	RRI, MS
24	carotol	57.57	1596	0.04	RRI, MS
25	phytone	65.276	1844	0.02	RRI, MS
26	hexadecanoic acid	68.54	1956	0.01	RRI, MS
Total identified				99.78	
Monoterpene hydrocarbons				29.59	
Oxygenated monoterpenes				69.44	
Sesquiterpene hydrocarbons				0.23	
Oxygenated sesquiterpenes				0.06	
Others				0.46	

**Table 2:** Spatial and contact repellency of cumin seed EO, cumin aldehyde, and other test materials, in comparison to DEET and transfluthrin, against adult female sand flies (*P. papatasi*).

	Mean Spatial Repellency (%) ± SEM						Mean Contact Repellency	
	**15 min**	30 min	60 min	90 min	120 min	180 min	Avoidance Ratios (*p*-Values)	Avoidance Frequency (Scores)
**Treatment**	**Concentration: 78.6 μg/cm^2^**							
*γ*-terpinene	22.0 ± 7.6 a	26.0 ± 2.5 a	37.9 ± 9.1 **a				**	0
*α*-pinene	32.6 ± 4.6 *a	29.9 ± 7.0 *a	34.3 ± 7.6 **a	22.1 ± 8.8 a	29.0 ± 9.5 *a	14.9 ± 10.0 a	***	0.2
*β*-pinene	39.3 ± 9.9 **a	37.2 ± 5.4 **a	10.3 ± 7.8 a	13.7 ± 17.6 a	50.9 ± 17.5 **a	25.6 ± 6.3 a	*	0.3
*β*-myrcene	13.8 ± 16.5 a	18.2 ± 21.0 ab	51.1 ± 13.2 ***ab	59.3 ± 0.70 **ab	75.0 ± 5.83 ***b	75.0 ± 5.83 ***b	*	0.1
*p*-cymene	54.7 ± 16.2 ***a	66.8 ± 12.1 ***ab	68.0 ± 17.9 ***ab	81.5 ± 9.8 ***ab	93.8 ± 3.3 ***b	97.5 ± 2.5 ***b	***	1
octanol	100 ± 0 ***a	100 ± 0 ***a	100 ± 0 ***a	100 ± 0 ***a	100 ± 0 ***a	100 ± 0 ***a	***	1
1-octen-3-ol	43.4 ± 7.6 **a	88.5 ± 5.8 ***b	97.2 ± 2.8 ***b	97.4 ± 2.6 ***b	100 ± 0 ***b	97.4 ± 2.6 ***b	***	1
DEET	25.4 ± 10.3 a	24.2 ± 19.4 *a	45.4 ± 12.4 ***ab	66.5 ± 12.0 ***b	80.6 ± 11.5 ***b	76.6 ± 13.7 ***b	***	1
**Concentration: 19.6 μg/cm^2^**								
cumin seed EO	100 ± 0 ***a	97.8 ± 2.2 ***a	100 ± 0 ***a	100 ± 0 ***a	100 ± 0 ***a	97.6 ± 2.4 ***a	***	1
cumin aldehyde	97.2 ± 2.8 ***a	100 ± 0 ***a	94.7 ± 5.2 ***a	75.0 ± 14.0 ***a	57.2 ± 11.7 ***ab	9.4 ± 26.5 b	***	0.97
**Concentration: 0.1572 μg/cm^2^**								
transfluthrin	32.8 ± 19.9 *a	24.4 ± 10.9 a	22.7 ± 7.4 a	20.6 ± 14.5 a	22.3 ± 2.9 a	53.4 ± 10.4 **a	***	0.7
**Control**								
	−7.2 ± 8.7 a	−13.7 ± 10.2 a	−5.0 ± 9.3 a	−1.8 ± 13.2 a	−4.8 ± 4.8 a	−11.0 ± 3.9 a		0

SEM: standard error of the mean (*n* = 4–7). Asterisks in spatial repellency columns indicate a significant difference (*p* ≤ 0.05 *; *p* ≤ 0.01 **; *p* ≤ 0.001 ***) between means of spatial repellencies of each treatment vs. control tubes at a given time point. One-way ANOVA with Dunnett’s test, *p* < 0.05; Asterisks in the contact repellency columns (*p*-values) indicate a significant difference between the mean avoidance ratios (calculated over a 3 h period based on six time points) for each treated compared to the untreated filter paper (located at the opposite end of the treatment in the static-air chamber), as well as compared to the control assay. This was adjusted for multiple observation time points (*n* = 6, over 3 h), averaged over a 3 h period (except for *γ*-terpinene, *n* = 3, over 1 h). Statistical significance was determined using Fisher’s Exact 2-tailed test, with *p* < 0.05. Avoidance frequency: Mean scores were calculated over a 3 h period (based on six time points). The number ‘0’ indicates contact by at least one fly, and ‘1’ indicates complete avoidance. Lowercase letters in the rows indicate a significant difference across the different time points for each treatment. LMM, with post hoc Sidak-adjusted test, *p* < 0.05. All treatments are present at 78.6 μg/cm^2^ concentration except cumin seed EO and cumin aldehyde at 19.6 μg/cm^2^, and transfluthrin at ~0.1572 μg/cm^2^.

**Table 3:** EC_50_ estimates of spatial repellency for cumin seed EO, cumin aldehyde, and octanol, compared to DEET and transfluthrin, against adult female sand flies (*P. papatasi*).

	EC_50_ (95%CI) μg/cm^2^, Slope, R^2^		
Treatment	15 min	30 min	60 min
cumin seed EO	0.97 (0.78–1.16), 3.92, 0.92	0.88 (0.68–1.08), 3.68, 0.90	0.34 (0.01–0.67), 1.90, 0.89
cumin aldehyde	0.15 (0.04–0.25), 0.96, 0.95	0.13 (0.07–0.18), 1.10, 0.96	0.07 (0.04–0.10), 2.44, 0.88
octanol	1.40 (0.67–2.12), 4.61, 0.97	0.85 (0.44–1.26), 3.14, 0.94	0.60 (0.30–0.91), 1.74, 0.91
DEET	90.72 (67.82–113.62), 6.32, 0.83	83.81 (31.79–135.83), 26.41, 0.94	78.95 (63.41–94.50), 13.48, 0.71
transfluthrin	0.06 (0.02–0.10), 1.83, 0.83	0.03 (0.02–0.04), 3.14, 0.86	0.04 (0.02–0.05), 4.37, 0.73

EC_50_, half-maximal effective concentration; CI, confidence interval; R^2^, coefficients of determination.

**Table 4:** Knockdown (KND ± SEM, %) effects of cumin seed EO, cumin aldehyde, and transfluthrin against adult female sand flies (*P. papatasi*).

Treatment	Concentration, μg/cm^2^	KND ± SEM, %					
		15 min	30 min	60 min	90 min	120 min	180 min
Cumin seed EO	157.2	0 ± 0 Aa	2.05 ± 1.2 Aa	8.3 ± 3.6 Aa	70.2 ± 12.7 Ab	100 ± 0 Ac	100 ± 0 Ac
	78.6	0 ± 0 Aa	0 ± 0 Aa	25.0 ± 16.0 Aa	30.8 ± 23.3 Aab	30.8 ± 23.3 Bab	96.2 ± 3.8 Ab
cumin aldehyde	78.6	0 ± 0 Aa	0 ± 0 Aa	25.0 ± 25.0 Aab	75.0 ± 25.0 Aab	63.8 ± 22.8 Aab	97.4 ± 2.6 Ab
	39.2	2.2 ± 2.2 Aa	0.00 Aa	2.3 ± 1.3 Aa	17.0 ± 9.0 ABa	63.8 ± 19.2 Ab	98.8 ± 1.2 Ab
	19.6	0 ± 0 Aa	0 ± 0 Aa	0 ± 0 Aa	0 ± 0 Aa	0 ± 0 Ba	57.3 ± 25.3 Ab
transfluthrin	7.86	43.1 ± 5.9 Aa	53.8 ± 2.7 Aab	59.8 ± 6.7 Aab	61.7 ± 2.6 Aab	59.7 ± 4.9 Aab	73.7 ± 2.4 Ab
	0.786	18.5 ± 8.5 Aa	16.8 ± 5.0 Ba	14.4 ± 5.6 Ba	10.4 ± 6.9 Ba	14.3 ± 10.7 Ba	9.7 ± 5.2 Ba

KND, knockdown; SEM, standard error of the mean (*n* = 4–6). Capitalized letters indicate significant differences across different concentrations within the same test substance (columns, one way ANOVA with Tukey’s HSD test, *p* < 0.05 (transfluthrin), or Kruskal–Wallis with Dunn’s test, *p* < 0.05 (cumin seed EO and cumin aldehyde)). Lowercase letters indicate significant differences across the different time points for each treatment (rows, LMM, with post hoc Sidak-adjusted test, *p* < 0.05).

## Data Availability

The original contributions presented in the study are included in the article/Supplementary Materials, further inquiries can be directed to the corresponding author.

## Supplementary Materials

The following supporting information can be downloaded at https://www.mdpi.com/article/10.3390/insects16060599/s1, Table S1. Concentrations of compounds tested in the static-air repellency bioassays and EC_50_ determination.
