# Supplementary material for: Investigation of Essential Oil from Cumin (Cuminum cyminum) Seeds and Selected Terpenes as Repellents Against Adult Female Phlebotomus papatasi (Scopoli) (Diptera: Psychodidae) Sand Flies
Source: Insects. 2025 Jun 6;16(6):599. doi: 10.3390/insects16060599 (PMC12193036; doi:10.3390/insects16060599)
Supplement: Supplementary file 1 [file insects-16-00599-s001.zip › insects-3601922-supplementary.pdf]

<u>Supplementary Material</u>

# Investigation of Essential Oil from Cumin (*Cuminum cyminum*) Seeds and Selected Terpenes as Repellents Against Adult Female *Phlebotomus papatasi* (Scopoli) (Diptera: Psychodidae) Sand Flies

**Maia Tsikolia [1], Panagiota Tsafrakidou [1,2], Michael Miaoulis [1], Andrew Y. Li [3], Dawn Gundersen-Rindal [3] and Alexandra Chaskopoulou [1,*]**

[1] European Biological Control Laboratory, USDA-ARS, 54 Marinou Antipa Str., 57001 Thessaloniki, Greece; mtsikolia@ars-ebcl.org (M.T.); panag.tsafrak@gmail.com (P.T.); mmiaoulis@ars-ebcl.org (M.M.)
[2] American Farm School, 54 Marinou Antipa Str., 57001 Thessaloniki, Greece
[3] Invasive Insect Biocontrol and Behavior Laboratory, USDA-ARS, 10300 Baltimore Avenue, Beltsville, MD 20705, USA; andrew.li@usda.gov (A.Y.L.); dawn.gundersen-rindal@usda.gov (D.G.-R.)
* Correspondence: achaskopoulou@ars-ebcl.org; Tel.: +30-2310492763

**Table S1.** Concentrations of compounds tested in the static-air repellency bioassays and $EC_{50}$ determination.

| Compound | Tested concentration (μg/cm²) | Solution concentration (%, w/v) |
|---|---|---|
| $\gamma$-terpinene | 78.6 | 0.5 |
| $\alpha$-pinene | 78.6 | 0.5 |
| $\beta$-pinene | 78.6 | 0.5 |
| $\beta$-myrcene | 78.6 | 0.5 |
| $p$-cymene | 78.6 | 0.5 |
| 1-octen-3-ol | 78.6 | 0.5 |
| octanol | 157.2 | 1 |
| | 117.9 | 0.75 |
| | 78.6 | 0.5 |
| | 39.2 | 0.25 |
| | 15.72 | 0.1 |
| | 3.92 | 0.025 |
| | 1.572 | 0.01 |
| | 0.392 | 0.0025 |
| | 0.1572 | 0.001 |
| DEET | 196.5 | 1.25 |
| | 157.2 | 1 |
| | 117.9 | 0.75 |
| | 78.6 | 0.5 |
| | 39.2 | 0.25 |
| | 15.72 | 0.1 |
| cumin seed EO | 157.2 | 1 |
| | 78.6 | 0.5 |
| | 39.2 | 0.25 |
| | 19.6 | 0.125 |
| | 1.572 | 0.01 |
| | 0.786 | 0.005 |
| | 0.392 | 0.0025 |
| | 0.1572 | 0.001 |
| | 0.0786 | 0.0005 |

| | | |
|---|---|---|
| cumin aldehyde | 78.6 | 0.5 |
| | 39.2 | 0.25 |
| | 19.6 | 0.125 |
| | 1.572 | 0.01 |
| | 0.786 | 0.005 |
| | 0.392 | 0.0025 |
| | 0.1572 | 0.001 |
| | 0.0786 | 0.0005 |
| | 0.01572 | 0.0001 |
| transfluthrin | 7.86 | 0.05 |
| | 0.786 | 0.005 |
| | 0.3144 | 0.0025 |
| | 0.1572 | 0.001 |
| | 0.0786 | 0.0005 |
| | 0.0392 | 0.00025 |
| | 0.01572 | 0.0001 |
